# Clinical characteristics and disease course before and after SARS-CoV-2 infection in a large cohort of systemic sclerosis patients

**DOI:** 10.55730/1300-0144.5768

**Published:** 2023-12-21

**Authors:** Aslıhan AVANOGLU GÜLER, Büşra ÖZÇİMEN, Mesude Seda AYDOĞDU, Alper SARI, Aliyeva NUMUNE, Nazife TÜZÜN ERSAN, Seda ÇOLAK, Hazan KARADENİZ, İbrahim VASİ, Hamit KÜÇÜK, Yasemin YALÇINKAYA, Abdülsamet ERDEN, Mehmet KAYAALP, Mehmet Akif ÖZTÜRK, Berna GÖKER, Ahmet OMMA, Sedat YILMAZ, Süleyman Serdar KOCA, Murat İNANÇ, Ali AKDOĞAN, Abdurrahman TUFAN

**Affiliations:** 1 Division of Rheumatology, Department of Internal Medicine, Faculty of Medicine, Gazi University, Ankara, Turkiye; 2 Department of Internal Medicine, Faculty of Medicine, Hacettepe University, Ankara, Turkiye; 3 Division of Rheumatology, Department of Internal Medicine, Faculty of Medicine, Fırat University, Elazığ, Turkiye; 4 Department of Rheumatology, Etlik City Hospital, Ankara, Turkiye; 5 Division of Rheumatology, Department of Internal Medicine, Faculty of Medicine, İstanbul University, İstanbul, Turkiye; 6 Department of Internal Medicine, Gazi University Hospital, Ankara, Turkiye; 7 Department of Rheumatology, Gülhane Training and Research Hospital, University of Health Sciences, Ankara, Turkiye; 8 Department of Internal Medicine, Yıldırım Beyazıt University, Ankara, Turkiye; 9 Department of Rheumatology, Ankara City Hospital, Health Sciences University, Ankara, Turkiye; 10 Division of Rheumatology, Department of Internal Medicine, Faculty of Medicine, Hacettepe University, Ankara, Turkiye; 11 Inflammatory Disease Section, National Human Genome Research Institute, Bethesda, Maryland, USA

**Keywords:** Systemic sclerosis, COVID-19, interstitial lung disease, outcome, respiratory support

## Abstract

**Background/aim:**

The objective of this study is to evaluate the clinical presentations and adverse outcomes of Coronavirus Disease 2019 (COVID-19) in patients with systemic sclerosis (SSc) and assess the impact of SSc features on the clinical course of COVID-19.

**Materials and methods:**

In this multicenter, retrospective study, SSc patients with COVID-19 were included. Clinical features of SSc, along with detailed COVID-19 data, were extracted from medical records and patient interviews.

**Results:**

The study included 112 patients (mean age 51.4 ± 12.8 years; 90.2% female). SSc-associated interstitial lung disease (ILD) was evident in 57.1% of the patients. The findings revealed hospitalization in 25.5%, respiratory support in 16.3%, intensive care unit admission in 3.6%, and a mortality rate of 2.7% among SSc patients with COVID-19. Risk factors for respiratory failure, identified through univariate analysis, included ILD (OR: 7.49, 95% CI: 1.63–34.46), ≥1 comorbidity (OR: 4.55, 95% CI: 1.39–14.88), a higher physician global assessment score at the last outpatient visit (OR 2.73, 95% CI: 1.22–6.10), and the use of mycophenolate at the time of infection (OR: 5.16, 95 %CI: 1.79–14.99). Notably, ≥1 comorbidity emerged as the sole significant predictor of the need for respiratory support in COVID-19 (OR: 5.78, 95% CI: 1.14–29.23). In the early post-COVID-19 period, 17% of patients reported the progression of the Raynaud phenomenon, and 10.6% developed new digital ulcers. Furthermore, progression or new onset of dyspnea and cough were detected in 28.3% and 11.4% of patients, respectively.

**Conclusion:**

This study suggests a potential association between adverse outcomes of COVID-19 and SSc-related ILD, severe disease activity, and the use of mycophenolate. Additionally, it highlights that having comorbidities is an independent risk factor for the need for respiratory support in COVID-19 cases.

## 1. Introduction

Coronavirus Disease 2019 (COVID-19), caused by severe acute respiratory syndrome-coronavirus-2 (SARS-CoV-2), manifests with a diverse range of presentations, spanning from asymptomatic cases to severe disease, which may progress to acute respiratory distress syndrome (ARDS), respiratory failure, and multiorgan failure [1]. The severity and prognosis of SARS-CoV-2 infection are well-established to be associated with factors such as older age, male sex, comorbidities, and immunocompromising conditions [2, 3]. The precise pathophysiological mechanisms underlying severe COVID-19 remain unclear; however, evidence suggests that an uncontrolled immune response, heightened systemic inflammation, endothelial activation, and vascular damage play roles in its pathogenesis, mirroring mechanisms observed in systemic sclerosis (SSc) and autoimmune diseases [4]. Immunosuppressants like glucocorticoids and biologics, commonly employed in the management of autoimmune and autoinflammatory rheumatic diseases, emerge as key treatment modalities for severe COVID-19 [5]. Consequently, there is a significant interest in exploring the association between autoimmune rheumatic disorders and COVID-19, given the overlapping aspects that include the utilization of immunosuppressive treatments and involvement of shared pathogenic pathways.

SSc is a chronic autoimmune connective tissue disease, characterized by complex pathogenic features, including inflammation, vasculopathy, and fibrosis. These elements often lead to progressive, irreversible tissue damage and organ failure [6]. Interstitial lung disease (ILD) is one of the major manifestations in both subsets of SSc, namely limited cutaneous SSc (lcSSc) and diffuse cutaneous SSc (dcSSc). Moreover, ILD frequently progresses to pulmonary fibrosis, representing the most common cause of SSc-related deaths, accounting for approximately 20% of overall mortality. In the context of SSc, infections are recognized as common contributors to mortality [7, 8]. Notably, infections occurring in the presence of ILD or pulmonary fibrosis are associated with an elevated risk of mortality [9, 10].

In SSc, endothelial activation and vascular injury represent key pathogenic mechanisms leading to persistent ischemia, clinically manifested as Raynaud phenomenon (RP), digital ulcers (DUs), and gangrene. Numerous clinical reports and autopsy-based studies have demonstrated that pulmonary fibrosis, endothelial activation, and vascular damage significantly contribute to the exacerbation and severity of COVID-19 [11, 12]. The available evidence suggests that clinical outcomes and the progression of SARS-CoV-2 infection may have detrimental effects on SSc patients. Furthermore, postinfection, clinical manifestations of SSc may deteriorate, particularly in patients with major organ involvement.

In this study, our primary objective was to evaluate the clinical manifestations and prognosis, including hospitalization, respiratory support, and complications, of SARS-CoV-2 infection in patients with SSc. Our secondary aims were to explore the impact of SSc characteristics and organ involvement on the course of COVID-19 and to assess the change in the clinical course of SSc following the occurrence of COVID-19.

## 2. Material and methods

### 2.1. Study design and participants

This study was a retrospective, observational, and multicenter investigation that enrolled SSc patients meeting the American College of Rheumatology/European League Against Rheumatism 2013 criteria from six referral hospitals [13]. The diagnosis of COVID-19 was established through the detection of SARS-CoV-2 nucleic acid via real-time-polymerase chain reaction (RT-PCR) from nasopharyngeal swab/sputum or based on suggestive symptoms accompanied by consistent chest computed tomography (CT) findings. SSc patients with a confirmed diagnosis of COVID-19 were included in the study across the six participating centers. Approval for the study was granted by the Ministry of Health and the Institutional Ethics Committee (No: 322), and informed consent was obtained from all participants.

### 2.2. Data collection

Demographic data, clinical characteristics of SSc, comorbidities, and treatments utilized were gathered through patient interviews and electronic medical records. Recorded clinical features included disease subsets, RP, disease duration (from the onset of the first non-RP symptom to the last evaluation), the last modified Rodnan skin score (mRSS), history of DUs, presence of DUs, musculoskeletal involvement (arthralgia, arthritis, joint contractures, or myositis), gastroesophageal involvement (presence of gastroesophageal reflux symptoms and evidence of esophageal dysmotility detected by esophageal manometry, barium esophagogram, or endoscopy), cardiac involvement (diastolic dysfunction, pericardial effusion, pericarditis, myocarditis, or arrhythmias), pulmonary arterial hypertension (PAH) (suspected on echocardiography and confirmed by right heart catheterization), scleroderma renal crisis, SSc-related ILD (presence of ILD on CT with a restrictive pattern on pulmonary function tests), and specific SSc autoantibodies (anti-centromere and anti-topoisomerase I).

Detailed COVID-19 data, encompassing clinical symptoms, treatment modalities, hospitalization, prolonged hospitalization (defined as a hospital stay exceeding 7 days), oxygen support, outcomes, complications, and the presence of long-COVID (defined as new or continued symptoms persisting for 4 weeks or more after the acute infection), were meticulously recorded [14]. The physician’s global assessment (PGA) of disease activity, measured on a numeric rating scale (NRS) ranging from 0 to 3 (indicating no severity to extremely severe disease), was recorded for the last visit prior to COVID-19 and the first visit after COVID-19. Additionally, patients were evaluated in terms of new-onset or progression of RP, DUs, dyspnea, and cough in the early post-COVID period, considered one month after the infection or hospital discharge to assess the impact of COVID-19 on SSc.

### 2.3. Statistical analysis

Data analysis was conducted using SPSS software v16.0 (SPSS Inc, Chicago, IL) and Microsoft Excel package programs. The distribution of variables was examined through visual methods such as histograms and probability plots, as well as analytical methods including Kolmogorov–Smirnov and Shapiro–Wilk tests. Depending on the distribution, results were reported using either the median with interquartile range (IQR) or the mean with standard deviation. Categorical variables were expressed as numbers and percentages. For analyses of categorical variables, the chi-squared or Fisher’s exact tests were applied. Group comparisons were performed using Student’s t-test, Mann–Whitney U test, or Wilcoxon tests, as appropriate. Regression analyses were performed to identify risk factors for respiratory support during the course of COVID-19, and odds ratios (ORs) with 95% confidence intervals (95%CI) were calculated. A p-value of less than 0.05 was considered statistically significant.

## 3. Results

The study included 112 SSc patients, with a predominant female composition (90.2%). The mean age of the patients was 51.4 ± 12.8 years, ranging from 24 to 89 years, and the median disease duration was 10.5 years. Almost half of the participants (48.2%) had at least one comorbidity, with hypertension being the most prevalent, followed by diabetes mellitus. Among the patients, 62.5% had lcSSc, and 57.1% had SSc-related ILD. Table 1 presents the baseline characteristics of SSc patients with COVID-19. The most commonly prescribed immunosuppressive treatments for SSc included low-dose glucocorticoids (52.7%) and mycophenolate (29.5%). Additionally, forty-one percent of patients were on hydroxychloroquine (Table 2).

Out of the total participants, 105 patients (93.7%) were diagnosed with COVID-19 through SARS-CoV-2 RT-PCR testing. Chest CT scans were conducted for 48 patients upon diagnosis, and two-thirds of these scans revealed findings consistent with COVID-19 pneumonia. The majority of patients (n = 96, 85.7%) exhibited typical symptoms of COVID-19. The most frequently reported symptoms were myalgia-arthralgia (55.4%), cough (48.2%), fever (47.3%), and dyspnea (33%). Notably, glucocorticoids were continued in 80% of patients, while mycophenolate was maintained in 30% throughout the course of COVID-19.

Patients received various COVID-19 treatments, including favipiravir (79.5%), oseltamivir (1.8%), low-dose aspirin (40.2%), low molecular weight heparin (31%), hydroxychloroquine (24.1%), glucocorticoids (16.1%), antibiotics (17%), tocilizumab (2.7%), colchicine (0.9%), and convalescent plasma (0.9%). Among them, 28 patients (25.5%) required hospitalization, with a median hospitalization duration of 9 days (min–max: 1–90 days) (Table 3).

Eighteen patients (16.3%) required respiratory support, with 58% receiving nasal oxygen. Invasive mechanical ventilation (IMV) was necessary for 4 patients (3.6%), and among them, three developed ARDS. Besides ARDS, observed complications related to COVID-19 in our cohort included acute renal failure, venous thromboembolism (extensive deep vein thrombosis and pulmonary embolism), dysrhythmias, and heart failure. The most prominent risk factor for developing complications was PAH (42.9% vs. 9.2%; p=0.031), followed by preexisting ILD and dcSSc (85.7% vs. 55.9% and 71.4% vs. 35%, respectively).

With the exception of one patient, all patients monitored in the intensive care unit (ICU) eventually died. Consequently, the COVID-19 mortality rate in SSc patients was determined to be 2.7%. The deceased patients were all under the age of 65 and had ILD and gastroesophageal involvement. Among them, two had dcSSc, and the remaining one had lcSSc with cardiac involvement. All were receiving mycophenolate treatment before contracting the infection. Notably, one patient with a normal immunoglobulin G level (1250 mg/dL) had received rituximab 4 months before the infection. The pre-COVID-19 PGA scores for all deceased patients were within the severe categories.

The comparison of patients concerning hospitalization revealed a higher prevalence of ILD and comorbidity among inpatients compared to outpatients (82.1% vs. 49.4%, p = 0.005; 67.9% vs. 42.7%, p = 0.037, respectively). Additionally, inpatients exhibited notably higher pre-COVID-19 PGA scores compared to outpatients (pre-COVID-19 PGA, median (IQR): 1(1) vs. 1(0), p = 0.016). Other clinical features were similar in both groups.

The assessment of clinical features and treatments in SSc patients requiring respiratory support demonstrated a significantly higher prevalence of ILD and comorbidities compared to SSc patients not requiring respiratory support (p = 0.008 and p = 0.01, respectively, Table 4).

Patients requiring respiratory support had more severe diseases (pre-COVID-19 PGA, median (IQR): 1(1) vs. 1(0), p = 0.01) and a higher prevalence of mycophenolate use before the infection (61.1% vs. 23.3%, p = 0.003). The identified risk factors for respiratory failure included the presence of ILD (OR = 7.49, 95% CI: 1.63–34.46, p = 0.01), having ≥1 comorbidity (OR = 4.55, 95% CI: 1.39–14.88, p = 0.01), higher pre-COVID-19 PGA (OR = 2.73, 95% CI: 1.22–6.10, p = 0.01), and the use of mycophenolate before the infection (OR = 5.16, 95% CI: 1.79–14.99, p = 0.003). Upon employing adjusted multivariate regression analysis, having ≥1 comorbidity emerged as the sole significant predictor of respiratory failure in COVID-19 (OR = 5.78, 95% CI: 1.14–29.23, p = 0.03) (Table 5).

Fifteen patients (13.4%) experienced prolonged hospital stays, and ILD was more prevalent in these patients compared to those with shorter hospital stays and outpatients (86.7% vs. 53.1%; p = 0.03). Long-COVID was observed in 3 patients (2.7%).

### 3.1. Clinical course of SSc patients after COVID-19

Pre- and post-COVID PGA scores were available for 72 patients. PGA scores increased in 15 patients shortly after COVID-19. The new-onset or progression of RP, DUs, dyspnea, and cough were individually reported. Eighteen patients (17%) had progression in RP, which was more frequent in males (27.8% vs. 6.9%, p = 0.020) and active smokers (22.2% vs. 5.7%, p = 0.045). Eleven patients (10.6%) developed new DUs after the infection, all of whom had a previous history of DUs, and five had active ulcers at the time of the infection. Anticentromere positivity was more common in patients with new DUs (54.5% vs. 22.5%, p = 0.032). The progression or new-onset dyspnea was reported in 30 patients (28.3%), two-thirds of whom (70%) had known SSc-related ILD. However, 20% of patients without baseline ILD had new-onset dyspnea. Twelve patients (11.4%) had new-onset or progression in cough, and it was associated with male sex and the use of Angiotensin II receptor blockers (ARBs) (male sex: 33.3% vs. 7.5%, p = 0.021; ARBs: 58.3% vs. 17.2%, p = 0.004). Worsening in PGA was associated with the progression or new onset of RP (p = 0.011), DUs (p = 0.023), and dyspnea (p = 0.003).

## 4. Discussion

The SARS-CoV-2 infection has become a critical health problem, causing severe outcomes and high mortality, particularly among individuals with chronic health conditions and those undergoing long-term immunosuppressive treatments [2, 3]. Given the shared pathogenetic mechanisms of COVID-19 and SSc [15], patients may face risks in two ways: SSc might negatively affect the course of COVID-19, and SARS-CoV-2 infection might accelerate underlying diseases by deteriorating preexisting involvements or leading to the emergence of new SSc manifestations. In the existing literature, only a few studies with small sample sizes have evaluated the impact and outcomes of COVID-19 in SSc patients, resulting in knowledge gaps in the management of SSc patients. This study aimed to fill these gaps by evaluating the clinical manifestations and outcomes of COVID-19 in a large sample of SSc patients and identifying adverse prognostic factors.

In our study, the majority of patients presented with typical COVID-19 symptoms, including myalgia-arthralgia, fever, and cough. The hospitalization rate was 25.5%, and it was more prevalent in patients with ILD, higher disease activity, and comorbidities. Additionally, the proportion of SSc-related ILD was significantly higher in patients with prolonged hospitalization.

A recent multicenter retrospective study demonstrated that ILD, PAH, and cardiac involvement, and rituximab use were associated with moderate-to-severe COVID-19 outcomes, including hospitalization and oxygen supplementation. The study identified the presence of ILD as the only independent predictor of adverse outcomes for COVID-19 [16]. The results from the European Scleroderma Trials and Research group (EUSTAR) study, which included 178 patients, indicated a hospitalization rate of 38% among SARS-CoV-2-infected SSc patients. Older age, comorbidities, renal disease, and ILD were significant characteristics associated with severe outcomes, including respiratory support and death. Furthermore, poor outcomes were observed in one-third of patients with preexisting ILD [17]. In SSc-ILD, older age, male sex, non-SSc comorbidities, and SSc-associated renal or cardiac disease were identified as factors associated with severe outcomes. Similarly, in our study, 16.3% of patients required respiratory support, and ILD was associated with respiratory failure in COVID-19. Comorbidity emerged as one of the independent predictors for the need for respiratory support in our cohort, a consistent finding observed in the general population. However, a significant association between specific comorbidities, including diabetes mellitus, hypertension, cardiovascular disease, and obesity, was not detected. High disease activity is considered a risk factor for COVID-19-related death in rheumatologic diseases [18], and our results supported this finding, as high disease activity prior to infection was identified as a risk for adverse outcomes. Controversial data exist in the literature regarding the effects of immunosuppressive treatments on COVID-19 [18, 19] outcomes. We did not find an association between the use of glucocorticoids or immunosuppressive agents such as rituximab and cyclophosphamide with hospitalization and respiratory failure in COVID-19. However, in univariate analyses, mycophenolate was significantly related to respiratory failure in our study.

Reported mortality from COVID-19 in SSc has varied from 5% to 12% in previous studies [16, 17]. Interestingly, the mortality rate in our cohort was 2.7%. Although SSc patients in our study shared similar characteristics with previous studies for most factors, they had a relatively younger age. Advanced age is considered a major risk factor for the severity and mortality of SARS-CoV-2 infection, which might explain the difference in mortality observed in our cohort [2].

In SSc, various viruses, including human cytomegalovirus, human herpesvirus-6, parvovirus B19, and Epstein-Barr virus, have been implicated in the worsening of SSc by activating endothelial activation/dysfunction and stimulating fibroblasts [20, 21]. Numerous studies have clarified that SARS-CoV-2 primarily targets the endothelium, which is a major pathway in the pathogenesis of SSc. This viral interaction leads to systemic vasculopathy, resulting in endothelial dysfunction, severe vascular injury, and thrombosis [22]. Furthermore, COVID-19 patients have demonstrated microvascular alterations and abnormalities in nailfold capillaroscopy [23, 24]. To date, two reported cases of SSc emerging after SARS-CoV-2 infection have been documented [25, 26]. Additionally, cases of SSc flaring have been reported after COVID-19 or SARS-CoV-2 mRNA vaccination [27, 28]. In our study, 21% of patients experienced an increase in disease activity after SARS-CoV-2 infection. In assessing vascular symptoms of SSc patients in the early post-COVID period, 17% of patients reported the exacerbation of RP, and 11% developed new DU. Another target of SARS-CoV-2 infection is the lung. The virus can lead to acute lung injury, inflammation, as well as epithelial injury, and this damage might eventually evolve into pulmonary fibrosis with myofibroblast proliferation and collagen deposition [29–31]. Therefore, SARS-CoV-2 infection might deteriorate or trigger lung involvement in SSc. As mentioned above, our results and previous reports have consolidated that the presence of ILD might influence the severity of COVID-19 in SSc. After COVID-19, new-onset or progression of dyspnea and cough were detected in 28% and 11.4% of patients, respectively. Interestingly, major organ involvements, including ILD, PAH, and cardiac involvement, were similar in both groups. However, 20% of patients without ILD had dyspnea, which is the most commonly reported symptom in post-COVID-19 syndrome [32]. Therefore, it is challenging to determine the relation of this manifestation based on COVID-19 or new lung involvement in SSc.

This study has several limitations. First, it is a retrospective study and lacks a control group. Second, due to the small number of adverse outcomes, such as death and complications related to COVID-19, we could not comprehensively evaluate the impact of SSc on these outcomes. Similarly, the frequencies of some important clinical involvements of SSc, like cardiac and renal involvement, and the use of specific immunosuppressive treatments (rituximab and cyclophosphamide), were low, potentially influencing our results. Additionally, we did not evaluate outcomes of COVID-19 with respect to SARS-CoV-2 variants. Despite these limitations, the major strength of this study lies in its large sample size of SSc patients with COVID-19, making it the first report to assess vascular, pulmonary, and overall disease activity of SSc patients after COVID-19.

## 5. Conclusion

In conclusion, this study indicates that ILD, comorbidity, and higher disease activity are associated with the hospitalization of SSc patients with COVID-19. Additionally, all of these clinical features, along with the use of mycophenolate, are identified as risk factors for respiratory failure in COVID-19. These findings suggest that, beyond preventive measures such as vaccination, controlling SSc disease activity may be crucial in protecting individuals from severe COVID-19.

## Figures and Tables

**Table 1:** Baseline characteristics of SSc patients with COVID-19 (n: 112).

Age, years (mean ± SD)	51.4 ± 12.8
Age ≥65 years, n (%)	16 (14.3)
Female, n (%)	101 (90.2)
Smoking, current/former/never, n (%)	9 (8)/18 (16,1)/85 (75.9)
Disease duration, median (IQR)	10.5 (11)
lcSSc/dcSSc, n (%)	70 (62.5)/42 (37.5)
mRSS, median (IQR)*	12 (10)
mRSS ≥15, n (%)*	18 (19.8)
RP, n (%)	102 (94.4)
History of DUs/current DUs, n (%)	56 (50.5)/14 (12.8)
Musculoskeletal involvement, n (%)	78 (69.6)
Arthralgia, n (%)	65 (58)
Arthritis, n (%)	15 (13.4)
Myositis, n (%)	3 (2.7)
Joint contracture, n (%)	14 (12.5)
Cardiac involvement, n (%)	14 (12.5)
Gastroesophageal involvement, n (%)	80 (71.4)
Renal crisis history, n (%)	2 (1.8)
PAH, n (%)	12 (10.7)
ILD, n (%)	64 (57.1)
FVC %, mean ± SD**	86.8 ± 18.6
FVC <%80, n (%) **	32 (36.8)
Anticentromere positivity, n (%)	27 (25)
Antitopoisomerase I positivity, n (%)	58 (54.2)

* mRSS was evaluated in 91 patients.

** FVC was evaluated in 87 patients before the infection.

DUs: digital ulcers; dcSSc: diffuse cutaneous systemic sclerosis; FVC: forced vital capacity; IQR: interquartile range; ILD: interstitial lung disease; mRSS: modified Rodnan skin score; PAH: pulmonary arterial hypertension RP: Raynaud phenomenon; SSc: systemic sclerosis.

**Table 2:** Comorbidities and current treatment of patients.

Comorbidities	n (%)
Diabetes mellitus	13 (11.6)
Hypertension	25 (22.3)
Cardiovascular disease	7 (6.3)
Chronic renal disease	3 (2.7)
Asthma	4 (3.6)
Malignancies	1 (0.9)
Obesity	6 (5.4)
Other comorbidities*	25 (22.3)
Comorbidity ≥1	54 (48.2)
**Treatment for SSc, n (%)**	
Hydroxychloroquine	46 (41.1)
Methotrexate	9 (8)
Glucocorticoids	59 (52.7)
Glucocoticoids ≥10 mg	5 (4.5)
Azathioprine	9 (8)
Mycophenolate	33 (29.5)
Cyclophosphamide	4 (3.6)
Rituximab	6 (5.4)
Vasoactive drugs	
CaCB	51 (45.5)
PDE5 inhibitors	18 (16.1)
ERA	7 (6.3)
Prostanoids	4 (3.6)
ACE-I	4 (3.6)
ARBs	26 (23.2)

* Other comorbidities include hypothyroidism, hyperlipidemia, atrial fibrillation, autoimmune hepatitis, and epilepsy.

ARB: Angiotensin II receptor blockers ACE-I: angiotensin-converting enzyme inhibitor; CaCB: calcium channel blockers; ERA: endothelin receptor antagonist; PDE5: phosphodiesterase 5.

**Table 3:** Outcomes and complications of COVID-19 in SSc patients.

Follow-up during COVID-19* n (%)	

Outpatient	82 (74.5)

Hospitalization	28 (25.5)
Duration of hospitalization, days (median, min–max)	9 (1–90)
Prolonged hospitalization >7 days	15 (13.6)

Need for respiratory support	18 (16.3)

ICU-IMV requirement	4 (3.6)

**Complications**, **n (%)** **	7 (6.5)

**Outcomes, n (%)**	

Recovery	106 (94.6)

Deceased	3 (2.7)

Long COVID	3 (2.7)

**Post-COVID period n (%)**	

New onset/worsening of RP (n = 105)	18 (17.1)

New onset DU (n = 104)	11 (10.6)

New onset/worsening of cough (n = 105)	12 (11.4)

New onset/worsening of dyspnea (n = 106)	30 (28.3)

* Follow-up information was assessed in 110 patients.

** Complications were evaluated in 108 patients.

DU: digital ulcer; ICU: intensive care unit; IMV: invasive mechanical ventilation; RP: Raynaud Phenomenon.

**Table 4:** The comparison of pre-COVID-19 characteristics of SSc patients according to need of respiratory support (n: 110).

	Respiratory support, No (n = 92)	Respiratory support, Yes (n = 18)	p
Age, years, mean ± SD	51.13 ± 12.94	53.28 ± 13.36	0.52
Age ≥65 years, n (%)	12 (13)	4 (22.2)	0.29
Sex, female n (%)	83 (90.2)	16 (88.9)	1.00
Disease duration, years (median, min–max)	10.5 (1–41)	10.5 (3–23)	0.79
Active smoking, n (%)	9 (9.8)	0	0.35
lcSSc/dcSSc, n (%)	60 (65.2)/32 (34.8)	9 (50)/9 (50)	0.34
mRSS, median (IQR)	11 (10)	12 (6)	0.30
mRSS ≥15, n (%)	15 (20.3)	3 (18.8)	1.00
ILD, n (%)	47 (51.6)	16 (88.9)	0.008
FVC, mean ± SD	87.39 ± 18.99	82.85 ± 17.09	0.42
FVC <80%, n (%)	25 (34.7)	6 (46.2)	0.53
PAH, n (%)	8 (9)	4 (22.2)	0.12
Cardiac involvement, n (%)	10 (11.2)	4 (22.2)	0.25
Gastroesophageal involvement, n (%)	66 (73.3)	13 (81.3)	0.75
Renal crisis history, n (%)	1 (1.1)	1 (5.6)	0.30
History of DU, n (%)	44 (44.8)	10 (55.6)	0.76
Active DU, n (%)	11 (12.2)	2 (11.1)	1.00
Anticentromere positivity, n (%)	24 (27.3)	2 (11.9)	0.23
Antitopoisomerase I positivity, n (%)	47 (54)	10 (55.6)	1.00
Comorbidity ≥1, n (%)	40 (43.5)	14 (77.8)	0.01
Hypertension, n (%)	21 (22.8)	4 (22.2)	1.00
Diabetes mellitus, n (%)	10 (10.9)	3 (16.7)	0.44
Cardiovascular disease, n (%)	5 (5.4)	2 (11.1)	0.32
Chronic renal disease, n (%)	2 (2.2)	1 (5.6)	0.42
Obesity, n (%)	4 (4.3)	2 (11.1)	0.25
Rituximab, n (%)	6 (6.6)	2 (11.1)	0.61
Mycophenolate, n (%)	21 (23.3)	11 (61.1)	0.003
Glucocorticoid, n (%)	45 (49.5)	13 (72.2)	0.13
Glucocorticoid ≥10 mg, n (%)	2 (4.4)	3 (23.1)	0.07
Cyclophosphamide, n (%)	4 (4.4)	0	1.00
Continuation of SSc specific treatment, n (%)	46 (52.9)	5 (31.3)	0.19
Pre-COVID-19 PGA, median (IQR)	1 (0)	1 (1)	0.01
Post-COVID-19 PGA, median (IQR)	1 (1)	1 (1)	0.32

DU: digital ulser; dSSc: diffuse systemic sclerosis; FVC: forced vital capacity; lSSc: limited systemic sclerosis; IQR: inrequartile range; ILD: interstitial lung disease; mRSS: modified Rodnan skin score; PAH: pulmonary arterial hypertension; PGA: physician global assessment.

**Table 5:** Risk factors for respiratory failure of COVID-19 in SSc patients according to univariate and multivariate analysis.

Variables	Univariate analysis		Multivariate analysis*	
	OR (95%CI)	p	OR (95%CI)	p
ILD	7.49 (1.63–34.46)	0.01	2.88 (0.45–18.46)	0.26
PAH	2.89 (0.77–10.91)	0.12	-	
Cardiac involvement	2.25 (0.62–8.21)	0.22	-	
Anticentromere positivity	0.33 (0.07–1.55)	0.16	-	
Comorbidity ≥1	4.55 (1.39–14.88)	0.01	5.78 (1.14–29.23)	0.03
Mycophenolate	5.16 (1.79–14.99)	0.003	3.22 (0.72–14.46)	0.12
Glucocorticoid	2.66 (0.87–8.06)	0.08	-	
Glucocorticoid ≥10 mg	6.45 (0.95–43.86)	0.05	-	
Pre-COVID-19 PGA	2.73 (1.22–6.10)	0.01	2.43 (0.88–6.73)	0.08

* In multivariate analysis adjusted for age and gender.

Incontinence interval: CI; ILD: interstitial lung disease; OR: odd ratio; PAH: pulmonary arterial hypertension; PGA: physician global assessment; SSc: systemic sclerosis.
